# Synthesis of short-range ordered aluminosilicates at ambient conditions

**DOI:** 10.1038/s41598-021-83643-w

**Published:** 2021-02-18

**Authors:** Katharina R. Lenhardt, Hergen Breitzke, Gerd Buntkowsky, Erik Reimhult, Max Willinger, Thilo Rennert

**Affiliations:** 1 Fachgebiet Bodenchemie mit Pedologie, Institut für Bodenkunde und Standortslehre, Universität Hohenheim, Emil-Wolff-Str. 27, 70599 Stuttgart, Germany; 2 Eduard-Zintl-Institut für Anorganische und Physikalische Chemie, Technische Universität Darmstadt, Alarich-Weiss-Str. 8, 64287 Darmstadt, Germany; 3 Institut für Biologisch Inspirierte Materialien, Universität für Bodenkultur Wien, Muthgasse 11/II, 1190 Wien, Austria

**Keywords:** Mineralogy, Environmental chemistry

## Abstract

We report here on structure-related aggregation effects of short-range ordered aluminosilicates (SROAS) that have to be considered in the development of synthesis protocols and may be relevant for the properties of SROAS in the environment. We synthesized SROAS of variable composition by neutralizing aqueous aluminium chloride with sodium orthosilicate at ambient temperature and pressure. We determined elemental composition, visualized morphology by microscopic techniques, and resolved mineral structure by solid-state ^29^Si and ^27^Al nuclear magnetic resonance and Fourier-transform infrared spectroscopy. Nitrogen sorption revealed substantial surface loss of Al-rich SROAS that resembled proto-imogolite formed in soils and sediments due to aggregation upon freezing. The effect was less pronounced in Si-rich SROAS, indicating a structure-dependent effect on spatial arrangement of mass at the submicron scale. Cryomilling efficiently fractured aggregates but did not change the magnitude of specific surface area. Since accessibility of surface functional groups is a prerequisite for sequestration of substances, elucidating physical and chemical processes of aggregation as a function of composition and crystallinity may improve our understanding of the reactivity of SROAS in the environment.

## Introduction

Sufficient release of aluminium (Al) and silicon (Si) by weathering of siliceous parent material facilitates precipitation of hydrous, short-range ordered aluminosilicates (SROAS). Accumulation of these phases results in coatings and alteromorphs in volcanic rocks^1,2^, clay-sized particles in andic soils^3–5^ or stream deposits from waters passing through extrusive rocks^6,7^. Formation of SROAS is not limited to volcanic parent material, but also takes place along pedogenetic transformation of more crystalline siliceous rocks^8–10^. The presence of SROAS imposes peculiar properties on soil material with respect to retention of plant nutrients^11^, pollutants^12^ and degradation products of soil organic matter (SOM)^13^, which promotes carbon sequestration in andic soils^14^. Efficient sorption of various compounds is ascribed to large surface areas and the microporous nature of SROAS, including the presence of reactive functional groups^15^. To investigate molecular interactions in detail, preparation of mineral analogues by synthesis is a common procedure in environmental research, since physical separation of SROAS from natural samples may not be sufficient with respect to mass or selectivity. For instance, synthetic SROAS were used to study their interactions with extracellular enzymes in soils^16,17^, adsorption mechanisms of potentially toxic metals^18,19^, and retention of organic and inorganic anions^20^.

Surface properties of SROAS are fundamentally related to their structure as it governs the quantity, reactivity and arrangement of functional groups. Microscopic techniques revealed that SROAS can develop distinct morphologies at the nanoscale, such as tubes and hollow spheres^21,22^. Tube-shaped SROAS have a well-defined structure and are termed imogolite^23^. Imogolite occurs as bundles of tubes with external diameters of approximately 2 nm that can reach lengths of several micrometres by anisotropic crystallization^8,21^. Its structure is built up by a dioctahedral Al sheet constituting the outer surface, with single Si tetrahedra linked to three Al octahedra inside the tube^23^. This specific configuration of Si can be detected by solid-state ^29^Si nuclear magnetic resonance (NMR) spectroscopy, henceforth denoted as Q^0^(3Al)^24^. Because of its morphology, imogolite offers porosity provided by pores with diameters < 1 nm^25^. Allophane is a generic term, referring to structurally less defined SROAS with variable chemical compositions that are classified according to their Al:Si ratio in the first instance^26^. Particularly Al-rich compounds (Al:Si ≥ 2) may contain Si in Q^0^(3Al) coordination and may be termed proto-imogolite, implying their function as precursors of more ordered phases^26^. As single hydroxyl groups coordinated to Al nuclei at defect sites are more reactive than bridging hydroxyls, adsorption capacity by ligand exchange decreases with crystallinity^27,28^. Given a sufficient Si supply, the Al:Si ratio decreases close to 1, and the atomic arrangement is more similar to tectosilicates, including Al in tetrahedral coordination (Al^IV^)^29^. As a result, particles exert permanent negative charge and exhibit fewer sites prone to ligand exchange due to a larger contribution of silanol groups^27,30^. Structural models that assume primary particles of both Al-rich and Si-rich SROAS to be hollow spheres have been proposed^31,32^, but have seldom been unambiguously verified in natural and synthetic samples. As crystallisation and assembly of precursors are likely inhibited in soil environments, respective SROAS may rather be perceived as fragments and poorly defined precursors of more ordered varieties^33^.

We aimed at synthesizing compounds that mimic pristine surfaces of SROAS forming at the initial stages of weathering. We thus employed ambient temperature and pressure conditions. Since organic species interfere with precipitation processes^34^ and may remain in the precipitate, we performed synthesis in aqueous media from inorganic salts of weakly complexing counterions. We achieved precipitation of SROAS by neutralizing an acid Al chloride solution with an alkaline sodium (Na) orthosilicate solution^35,36^. To obtain precipitates with different Al:Si ratios, we varied the input of Si to give molar concentration ratios of 1, 1.5 and 2. We used a monomeric Si source to favour the formation of Si in Q^0^(3Al) coordination and to minimize the inheritance of amorphous silica (SiO_2(am)_), but worked at decimolar concentrations, potentially enabling its formation. We report here on the structure of synthesized SROAS, studied by X-ray diffractometry (XRD), Fourier-transform infrared (FTIR) and solid-state ^27^Al and ^29^Si NMR spectroscopy, and its consequences on specific surface area (SSA) of dry solids because we consider them relevant for future syntheses and possibly the behaviour of similar minerals in soils and sediments. Furthermore, we describe the effects of cryomilling on the physical and structural properties of SROAS.

## Results and discussion

### Morphology and composition of SROAS

Elemental analysis of SROAS revealed that an increase in Si supply successfully led to advanced Si incorporation into the solids, resulting in Al:Si ratios of 2.6, 2.1 and 1.4 in the precipitates (Table 1). Silicon incorporation into the solid was accompanied by retention of Na, as observed by Na contents of 0.3, 1.8, and 23.1 mg g^−1^ with decreasing Al:Si ratios. This points to different amounts of negatively charged sites to be balanced by Na. In the following, we will refer to SROAS according to the classification in Al-rich (Al:Si ≥ 2) and Si-rich (Al:Si ≤ 2)^26^, giving the Al:Si ratio in parentheses to identify an Al-rich compound. All solids exhibited larger Al:Si ratios than those originally employed in synthesis batches, evidencing preferential loss of Si during dialysis. Relative to the amount of Si expected with complete incorporation into the solid and calculated from Al content and initial Al:Si ratios, the amount of Si in the precipitates was lower by 22–30%. This corresponds to a portion of 7–19 mmol Si l^−1^ in the retentate, which is a cautious estimation under the assumption of no Al loss. This value is well beyond the solubility of SiO_2(am)_ at pH 7^37^. Provided an excess of Si species was unaffected by the presence of Al, the kinetics of Si polymerization allow formation of SiO_2(am)_ within the timeframe of neutralization^38^. Depolymerization rates of SiO_2(am)_ are also sufficient to consider its dissolution during dialysis^39^. However, incomplete incorporation of Si into SROAS at ambient synthesis conditions but concentrations below 2 mM Si was reported previously and shown to depend on the Al:Si ratio, indicating Al speciation to be a controlling factor in Si removal^40^. Furthermore, it was shown that Si-rich SROAS are less stable, leading to a structure-dependent preferential mobilisation of Si^41,42^. Overall, it is thus very likely that Si was removed from the retentate both by diffusion of Si species that were not incorporated into the solid during neutralization and by the dissolution of unstable Si species during dialysis.

**Table 1:** Selected chemical and physical properties of synthesized short-range ordered aluminosilicates.

Initial Al:Si	Al:Si	Al	Si	Na	Si in Q^0^(3Al)	^IV^Al	^V^Al	^VI^Al	SSA	V_T_	V_2-10_	V_10-50_
		(mg g^−1^)			(% Si)	(% Al)			(m^2^ g^−1^)	(mm^3^ g^−1^)		
1	1.4	200.9	146.3	23.1	9	29	5	66	295.5			
*Cryomilled*									274.2	943	167	617
1.5	2.1	245.2	122	1.8	38	13	6	80	25.6			
*Cryomilled*									43.2	125	47	32
2	2.6	250.1	100.7	0.3	48	9	6	85	0.7			
*Cryomilled*					44	7	6	87	6.9	58	3	20

Proportion of Si nuclei in imogolite-like coordination (Q^0^(3Al)) and Al nuclei in tedrahedral (Al^IV^), pentahedral (Al^V^) and octahedral (Al^VI^) coordination was quantified by solid-state nuclear magnetic resonance spectroscopy. Specific surface area (SSA), total pore volume (V_T_) and pore volume of pores with diameters from 2–10 nm (V_2–10_) and 10–50 nm (V_10–50_) was derived from nitrogen sorption analysis.

**Figure 1:**
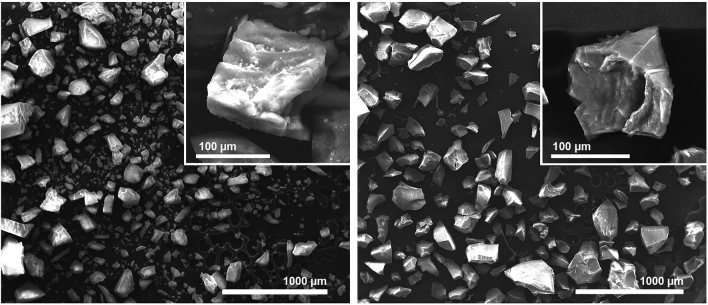
Environmental scanning electron microscopy images of Si-rich (Al:Si 1.4, left) and Al-rich short-range ordered aluminosilicates (Al:Si 2.6, right). The images were taken by Christian Buchmann.

Dry SROAS differed markedly in bulk density, whereas Si-rich SROAS occupied a much greater volume per mass than Al-rich SROAS (Supplementary Fig. S1). Visualized by environmental scanning electron microscopy (ESEM), all variants appeared as micrometre-sized angular particles with visible breaking edges (Supplementary Fig. S2). The SEM images show that Al-rich SROAS (2.6) consisted by trend of larger particles than Si-rich SROAS and aggregate surfaces of Si-rich SROAS appeared rougher (Fig. 1). Both Al-rich SROAS appeared to have smoother aggregate surfaces than Si-rich SROAS (Supplementary Fig. S3). Similar composition-dependent morphological differences were previously described as glassy versus granular aggregates and related to the mineral structure of precipitates^43^.

### Mineral structure of SROAS

**Figure 2:**
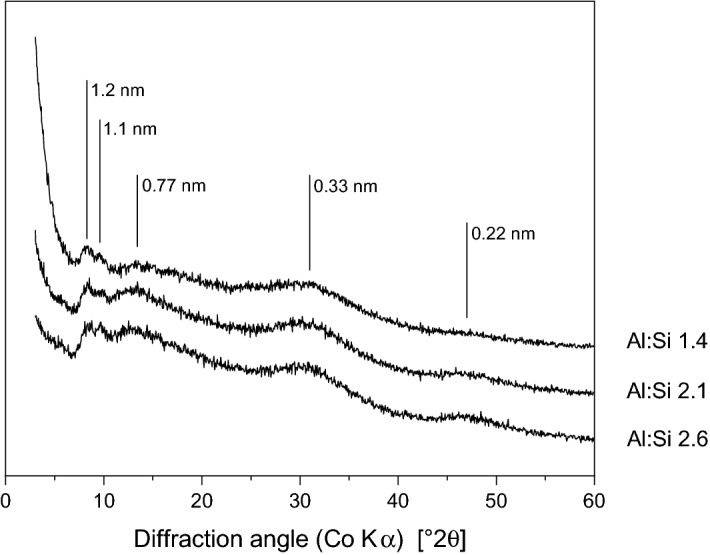
X-ray diffractograms of short-range ordered aluminosilicates. Data is shifted along the y-axis for clarity and given in arbitrary units.

All materials showed very broad reflections when exposed to Co Kα radiation indicative of a poorly ordered structure (Fig. 2). Reflections centred at *d*-values of 0.77, 0.33 and 0.22 nm are related to the unit cell of imogolite and were reported in several types of SROAS^23,26^. In Al-rich SROAS, reflections close to 0.77 and 0.22 nm were slightly more pronounced than in Si-rich SROAS (see Supplementary Fig. S4), pointing to a structural similarity to proto-imogolite of the former. Reflections at *d*-values above 0.9 nm were previously shown to be related to the extent of assembly of imogolite-like precursors^44^. Hence, poor resolution in this range evidences that structural repetition occurred at very narrow distances in the precipitates only.

**Figure 3:**
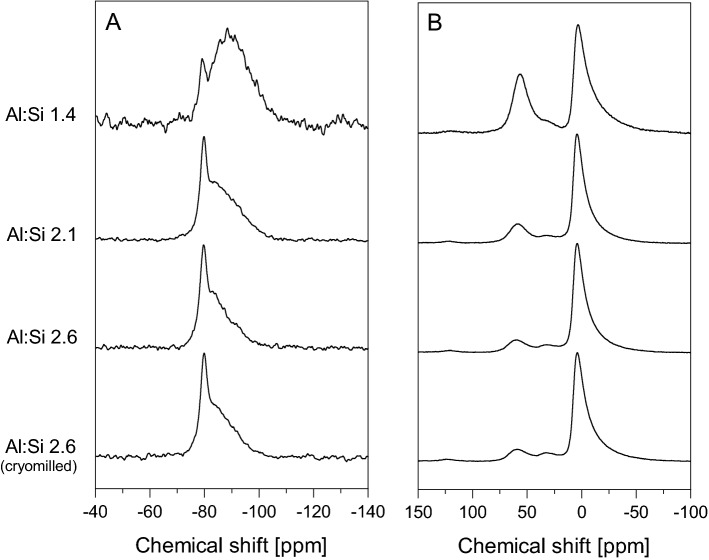
Solid-state nuclear magnetic resonance spectra (**A** ^29^Si; **B** ^27^Al) of short-range ordered aluminosilicates at a line broadening of 50 Hz.

All SROAS caused a symmetric peak centred at around -79 ppm in ^29^Si-NMR spectra indicative of Si in Q^0^(3Al) coordination^24^ (Fig. 3A). The proportion of Si nuclei in this chemical environment varied from 9% in Si-rich SROAS to 38% and 48% in Al-rich SROAS with Al:Si ratios of 2.1 and 2.6, respectively (Table 1). The presence of Q^0^(3Al) shows that oxolation of Al and Si precursors led to locally defined structural domains, but as the kinetics of this process are poorly resolved, it is not clear whether it occurred during the neutralization step or by structural rearrangement during dialysis. Precipitates that formed within one hour by neutralization at ambient conditions at decimolar concentrations analysed previously contained Si in Q^0^(3Al) coordination together with ill-defined Si environments, indicating that crystallization is initiated rapidly^35,44^. We did not observe any signals at positions above -79 ppm^45^, demonstrating that small polynuclear species that may have formed during the initial steps of condensation reactions were consumed prior to structural analysis. At more negative chemical shifts, resonances of ill-defined Si species appeared in the spectra. Condensation of Si tetrahedra causes shielding of Si nuclei and thus upfield chemical shifts, whereas the extent depends on the number of substituted silanol groups^46^. However, the upfield shift is counteracted by reactions with Al, since the deshielding effect of Al leads to a downfield shift with an increasing number of bonds between Al and Si^46^. The intensity distribution between − 80 and − 110 ppm differed among SROAS, whereas the region at higher chemical shifts was favoured in Al-rich SROAS. This indicates that Si in ill-defined coordination comprised more bonds to Al nuclei on average in Al-rich SROAS. In Si-rich SROAS, bonds between Si tetrahedra contributed, but the amount of Si nuclei bound to four Si tetrahedra was low as indicated by only small spectral intensities at chemical shifts below − 95 ppm^46^. Consistent with previous reports, we found that the ratio of Si nuclei in Q^0^(3Al) coordination to nuclei in more ill-defined species decreased with Si input^47^. The ill-defined fraction was observed to be lost upon heating, indicating its lower stability and transformation in favour of more ordered phases^35,44,48^.

Solid-state ^27^Al NMR spectra of SROAS exhibited two main peaks attributable to Al^IV^ and Al nuclei in octahedral coordination (Al^VI^; Fig. 3B)^49^. The latter was detected by an asymmetric peak exhibiting its maximum at 4 ppm in Al-rich SROAS, the intensity of which constituted 80–85% of the signal (Table 1). In Si-rich SROAS, this peak was slightly shifted upfield to 3 ppm, and its intensity contribution was lower, amounting to 66%. The chemical shift of Al nuclei is not only affected by the number of adjacent oxygen atoms but also sensitive to the structural arrangement of coordination spheres. This can be exploited to conclude the state of the dioctahedral sheet. Octahedral Al in well-crystallized gibbsite resonates at 9.5 ppm, which is shifted upfield with structural disorder to 8.5 ppm in X-ray amorphous Al hydroxides^50^. Octahedral Al in synthetic imogolite with well-developed tubular morphology exhibits peak maxima at 4–6 ppm^44,48^. Both synthetic imogolite precursors and natural samples of proto-imogolite obtained from weathered volcanic ejecta contain Al^VI^ resonating at 4–6 ppm^29,33,51^. Low chemical shifts of Al^VI^ nuclei in Al-rich SROAS thus corroborate results derived from XRD. We conclude that assembly of polynuclear species with a local imogolite-like Si environment did not lead to spatially ordered tube- or ball-shaped particles during synthesis, but rather small, likely curved, fragments^44,48^.

Both Al- and Si-rich SROAS contained Al^IV^ as observed by peaks centred at 56–60 ppm. The contribution of Al^IV^ increased with Si content from 9 to 13% in Al-rich SROAS and a further surge to 29% in Si-rich SROAS (Table 1). Compared with natural samples from altered volcanic ejecta, contents of Al^IV^ in Al-rich SROAS were in the same range, but Si-rich SROAS contained more than natural analogues previously characterized (max. 21%)^29^. A small resonance line at 32–34 ppm indicated traces of Al in pentahedral coordination (Al^V^)^52^. Integration of spectra yielded an area contribution of about 5% for all three SROAS, but quantification is ambiguous due to poor peak separation particularly with Si-rich SROAS^52^. Chemical shifts of Al^IV^ in our precipitates resonated in the same range as observed in natural SROAS samples^29^, and its quantity is related to the amount of Si in ill-defined species as observed previously in natural and synthetic samples^47,51^. As Al^IV^ in tectosilicates and glasses resonates in the same range, this points to its incorporation to a network with Si tetrahedra^29^. Besides, transient species that form during neutralization of Al salts and contain Al^IV^ resonating at 62–65 ppm can be inherited by a small extent into solid poorly ordered Al phases synthesized at ambient conditions^50,53,54^. Such species may also form during synthesis of SROAS and may be located at the edges of fragments of imogolite precursors^48^. The downfield shift with Al content may thus indicate corresponding structural variations.

**Figure 4:**
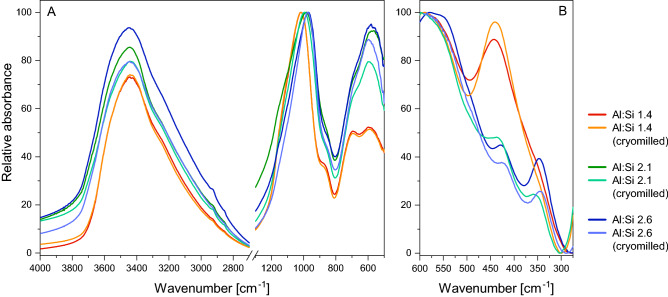
Fourier-transform infrared spectra of short-range ordered aluminosilicates recorded in transmission mode.

Infrared spectra of all SROAS show a broad absorption band centred at 3440 cm^−1^ due to OH stretching in hydroxyl groups and at 1640 cm^−1^ due to OH bending in adsorbed water (Fig. 4A and Supplementary Fig. S5). There was no difference in shape or position between SROAS. An absorption band between 1018 and 975 cm^−1^ is caused by stretching vibrations of oxygen bridges formed by condensation of Si and Al^55^. This band had the highest intensity for all materials. The position of the band shifted to higher wavenumbers with increasing Si content due to its sensitivity to the coordination environment of oxygen atoms^56,57^, corroborating the observation by ^29^Si NMR spectroscopy that the portion of Al–O–Si bonds increased with Al content relative to the portion of Si–O–Si bonds. All SROAS exhibited an absorption band between 590 and 570 cm^−1^ due to Al–OH bending vibrations^55^. Relative to the intensity of the band in the Si–O–(Al) stretching region, this band decreased in intensity with increasing Si content as observed previously^56^. A shoulder around 870 cm^−1^ may be related to Al–OH bending or Si–OH stretching^23^. Characterization of Si-rich SROAS revealed an absorption maximum at 690 cm^−1^. Regarding Al-rich SROAS, this band only occurred as a shoulder and its intensity was lower relative to Al–OH bending at 590–570 cm^−1^. This corresponds to a trend previously observed in SROAS with increasing Si content and is caused by differences in coordination environments of OH groups related to Al and Si speciation^56,57^. In contrast to Al-rich (2.6) SROAS, Si-rich SROAS did not cause a distinct band at 348 cm^−1^ indicating that the concentration of Q^0^(3Al) was too low to be detected by FTIR (Fig. 4B)^56^. Both phases showed a band at 430–440 cm^−1^ related to vibrations of silanol groups, the intensity and frequency of which increased with Si content as observed previously^56^.

Synthesis at high batch volumes and rapid neutralization may favour structural inhomogeneity caused by local pH gradients^45^, but we did not observe any indication of Al hydroxides or amorphous silica. Crystalline Al hydroxides are detectable by XRD and FTIR, but we did not observe any distinct corresponding feature^58^. However, since poorly crystalline Al hydroxides may give no reflections in XRD^50,58^ and reflections in our diffractograms were poorly resolved, we cannot exclude their formation. Aluminium-rich SROAS did not contain polymerized Si tetrahedra as derived from ^29^Si NMR spectra, whereas a pure SiO_2(am)_ phase is hardly differentiable from aluminosilicate polymers in Si-rich SROAS.

### Physical properties as derived from nitrogen sorption

We studied N_2_ sorption to SROAS to derive SSAs by the BET method and found distinct differences between compositional varieties. Nitrogen sorption increased with Si content and Si-rich SROAS held the greatest SSA of 295.5 m^2^ g^−1^ with a C value of 141 (Table 1; Supplementary Table S1). Both parameters decreased in Al-rich (2.1) SROAS to a SSA of 25.6 m^2^ g^−1^ and a C value of 110. Nitrogen sorption to Al-rich (2.6) SROAS was surprisingly low. A specific surface area of 0.7 m^2^ g^−1^ and a C value < 10 was derived from linear regression of the BET equation but the magnitude of N_2_ sorption constrains measurement accuracy^59^. Assuming mineral material to be a monodisperse assemblage of smooth cubic particles, SSA is geometrically related to the edge length by six divided by density [g cm^−3^] times edge length [µm]^60^. Densities of natural and synthetic SROAS with variable Al:Si ratios have been reported to range between 1.8 and 2.7 g cm^−3^^61^. It follows that SSA is driven by the mass fraction of particles with an edge length < 1 µm. As assessed from particle visualization by ESEM, the mass fraction of particles < 1 µm was negligible in both Al-rich (2.6) SROAS and minor in Si-rich (1.4) SROAS. Hence, N_2_ sorption data of Al-rich (2.6) SROAS corresponded well with microscopic characterization and suggested that the solid consisted of dense aggregates that were not penetrated by N_2_ molecules under measurement conditions. On the other hand, particle sizes of Al-rich (2.1) and particularly Si-rich (1.4) SROAS were not sufficient to explain the higher amount of sorbed N_2_. This leads to the conclusion that either surface roughness at the submicron scale or intraaggregate porosity advances N_2_ sorption to these materials.

Synthetic SROAS can exhibit SSA of up to 700 m^2^ g^−1^, but aggregation is a common process restricting surface accessibility of primary particles for N_2_ molecules and thus frequently resulting in lower values^36,44,57^. However, very low N_2_ sorption with SSA below 5 m^2^ g^−1^ is rather rare, but has been previously reported^44,57,62–64^. Notably, and in accordance with our results, such a massive surface reduction did only occur in precipitates with an Al:Si ratio close to 2 and poor spatial order, indicating that this phenomenon is specific of a certain composition. Precipitates synthesized at ambient temperature by Du et al.^44^ were structurally very similar to our Al-rich (2.6) SROAS and only developed microporous nature and greater SSA upon assembly to tile- and ultimately tube-shaped particles induced by heating. Crystallization was likely inhibited in synthesis protocols that previously reported exceptionally low SSA^44,57,62–64^ either by decimolar concentrations^45^, the presence of counterions during the heating step^65^ or ambient synthesis conditions^44^.

As precipitation by neutralization yielded turbid suspensions of small particles, aggregation resulting in micrometre-sized particles was obviously provoked during the dehydration procedure. Drying conditions varied between synthesis protocols that previously observed very low SSA of Al-rich SROAS^44,57,62–64^ and included freeze-drying^44,57,64^ and drying at 110 °C^63^ or were not specified^62^. Outgassing of the samples before N_2_ sorption measurements was done at temperatures from 100 to 400 °C, whereas we worked at T = 80 °C, since structural changes were reported at T > 100 °C^66^. Crystallization of poorly ordered Al and iron (Fe) phases is accompanied by a substantial loss of SSA and is possible at T < 100 °C^67,68^. Although SROAS rearranged structurally upon heating to T = 100 < 400 °C, coinciding changes in SSA were small^69,70^, indicating that the magnitude of SSA is imposed on the dry solid beforehand. Indeed, we observed that thawing of frozen synthesis batches yielded clear suspensions with rapidly settling particles and thus assumed that aggregation happened primarily during the freezing process. We repeated N_2_ sorption analysis of Al-rich (2.6) SROAS obtained by decanting, drying and outgassing at 40 °C. The N_2_ sorption capacity of decanted aggregates was not substantially larger (calculated SSA = 1.9 m^2^ g^−1^, C < 10, Supplementary Table S1). We thus conclude that processes during freezing determined the physical properties of the dry mineral material.

Formation of dense aggregates from aqueous suspensions of Al and Fe oxides and hydroxides upon freezing was previously observed^71^ and can be explained by physical interactions of ice, liquid water and suspended particles^72,73^. As we froze large volumes (400 ml) of suspension at − 20 °C, cooling was limited by heat conductance of ice and water and ice formation progressed only slowly from the exterior of the volume inward (ca. 0.6–1.4 µm s^−1^)^71^. At such low freezing rates, particles with a diameter < 1 µm are expelled from the freezing front and transported^72^. This phenomenon is caused by repulsive forces caused by surface tension of a film of liquid water between the freezing front and the particle^72^. The co-occurrence of ice and liquid water induces migration of the latter to the former, usually referred to as cryosuction. When suspensions with sufficient particle concentration are slowly frozen, the combination of particle repulsion and cryosuction leads to a concentrated layer of particles ahead of the freezing front^73,74^. As this layer freezes, colloidal suspensions are segregated on a macroscopic scale into areas with crack-like boundaries between ice and particle enriched zones^73,74^. Similar segregation phenomena are likely responsible for the shape of SROAS aggregates we observed by ESEM as sublimation of ice led to the disintegration of frozen batches. Stress caused by cryosuction and freezing has been observed to be sufficient to overcome electrostatic repulsion between particles and cause particle packing into clusters that do not disintegrate after thawing^71,74,75^. Interfacial effects reducing the mobility of water molecules and inhibiting complete crystallization^76^ ultimately limit the dehydration of poorly crystalline solids. Consistently, porosity analysis of Fe (hydr)oxide aggregates indicated that crystallites were separated by a hydration layer^71^. The magnitude of SSA of Al-rich SROAS and the fact that it is not substantially affected by outgassing evidence that inaccessibility of interparticle spaces to N_2_ molecules was caused by freezing and homogeneously distributed within the material since otherwise water emission would have created porosity. Small pore sizes and possibly sorbed water likely restricted N_2_ diffusion, an effect that is particularly relevant for pores < 1 nm^77^. Taking this effect into account by assuming a maximal pore radius of 2 nm, calculation of the respective particle diameter that is necessary to explain impermeable aggregate surfaces on the basis of cubic close-packing of monodisperse spheres gives a value of 4.8 nm (see Supplementary information). Considering the hydration layer (0.9–2 nm)^71^, this calculation illustrates that primary building entities have to be < 4 nm as structural analysis already indicated. Consistent with previous reports, we observed an increase in SSA with Si content in poorly ordered SROAS^57,63,78^, pointing to a structure-dependent effect on the spatial arrangement of mass at the submicron scale. In summary, increasing incorporation of Si into poorly ordered SROAS or advanced crystallization of Al-rich SROAS seems to cause resistance to compaction and consequently a rise in SSA.

Considering mechanisms and forces responsible for SROAS aggregation, Wells and Childs^79^ proposed a model based on the assembly of hollow spheres that recognized the degree of water removal as a measure to quantify the interactions between particles. As long as the hydration layer prevails around particles, van der Waals forces and electrostatic interactions are perceived to be responsible for holding particles together^79^, with a possible contribution of aluminol-group condensation^80^. The latter is especially likely during the formation of Al-rich SROAS in our syntheses by concentration of small Al-rich fragments. Our results show that incorporation of Si interferes with aggregation, but the mechanisms are not clear yet. Both the presence of silanol groups and oxygen bridges between Si tetrahedra as well as the formation of Al^IV^ imposes changes on surface properties that may lead to variations in interfacial processes between particles and water dynamics.

### Effects of cryomilling on particle size, mineral structure and SSA

**Figure 5:**
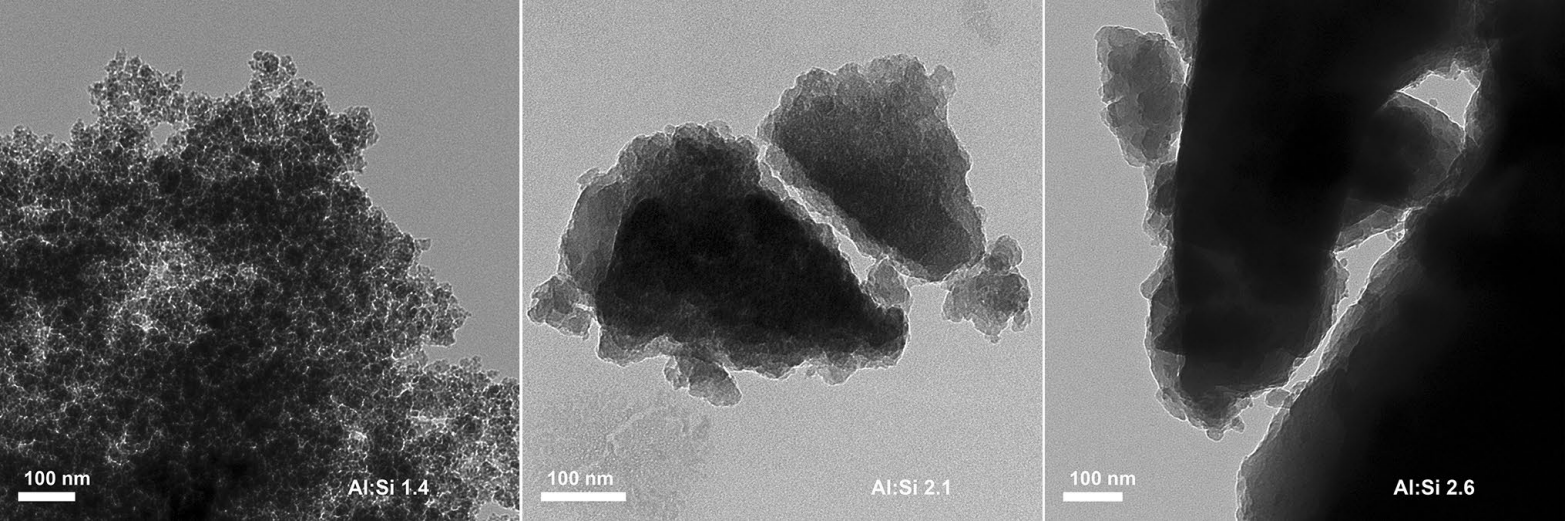
Transmission electron microscopy images of cryomilled Si-rich (Al:Si 1.4, left) and Al-rich (Al:Si 2.1 and 2.6, middle and right, respectively) short-range ordered aluminosilicates.

We aimed at increasing the yield of particles with diameters < 1 µm and greater SSA of mineral material and thus decided to mill SROAS. It was previously shown that SROAS are sensitive to heating and mechanical stress, resulting in structural changes^66,81^. Hence, we employed mechanical treatment of dry material under constant cooling with liquid N_2_, i.e. cryomilling. Cryomilling of metallic powders was shown to accelerate fracturing of particles but suppress structural rearrangement^82^. We investigated particle size and morphology in resulting powders by transmission electron microscopy (TEM). We did not find particles with diameters > 15 µm, indicating that cryomilling efficiently crushed aggregates already after 2–5 min (Supplementary Fig. S6). We found striking differences in particle morphology between Si-rich and Al-rich SROAS (Fig. 5). Both Al-rich SROAS assembled into aggregates of variably sized, dense particles with defined boundaries. On the contrary, Si-rich SROAS appeared as finely differentiated clusters, which could be observed as loose aggregates on the TEM carbon grid but rarely as particles with defined barriers. Many Al-rich SROAS particles with a diameter > 300 nm had an angular shape probably retained from disruption of primary material (Supplementary Fig. S7).

Inspection for structural rearrangement by FTIR spectroscopy of cryomilled samples revealed only small changes. Neither spectra showed a shift in the position of stretching vibrations of Si–O–Al bridges (1018–975 cm^−1^), but spectra of both Al-rich SROAS exhibited a small relative decrease of absorption by Al–OH bending at 595 cm^−1^ (Fig. 4A). At lower wavenumbers, absorption at 348 cm^−1^ in Al-rich SROAS (2.6) decreased, while absorption at 440 cm^−1^ in Si-rich SROAS increased (Fig. 4B). Hence, the spectra point to small changes of Si speciation induced by cryomilling, which led to a particular interest in the behaviour of Si in Q^0^(3Al) coordination. We thus repeated solid-state NMR spectroscopic analyses of Al-rich (2.6) SROAS, showing that Si in Q^0^(3Al) coordination was largely retained after cryomilling, but small amounts of more ill-defined species were generated by mechanical treatment in spite of low temperatures (Fig. 3A, Table 1). We did not try to resolve such changes in Si-rich SROAS, since ^29^Si NMR spectroscopy yielded a poor resolution of Si speciation in the primary material.

**Figure 6:**
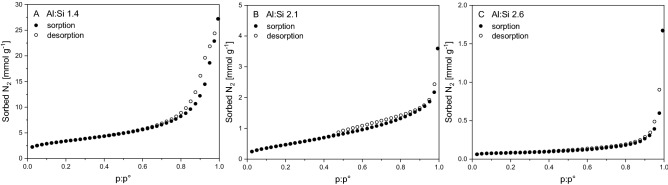
Nitrogen sorption and desorption isotherms at − 196 °C of cryomilled short-range ordered aluminosilicates with varying Al:Si ratios (**A** 1.4; **B** 2.1; **C** 2.6).

In addition to particle visualization by TEM, we employed N_2_ sorption experiments over the whole range of relative pressure to derive SSA and porosity of cryomilled SROAS. Silicon-rich (1.4) SROAS remained to be the strongest N_2_ sorbent after cryomilling. The measured sorption capacity at relative pressures below 0.35 was slightly lower compared to unmilled solid, resulting in a decrease of SSA by 7.2% (Table 1), but effects of this size might be related to measurement inaccuracy. We observed desorption hysteresis at relative pressures above 0.7 (Fig. 6A), indicating the presence of mesopores with diameters from 2 to 50 nm^83^. Evaluation of N_2_ sorption data with respect to micropore volume suggested negligible contribution of pores in this size range (< 2 nm)^59^. Nitrogen sorption to Al-rich SROAS increased after cryomilling, causing enhanced SSA (43.2 and 6.9 m^2^ g^−1^ for solids with Al:Si ratios of 2.1 and 2.6, respectively; Table 1). None of the sorption branches exhibited a distinct knee indicating completion of the monolayer and there was thus no evidence of accessible micropores (Fig. 6B,C). For Al-rich (2.1) SROAS, we detected desorption hysteresis between relative pressures of 0.45 and 0.9 evidencing present mesopores to have smaller diameters on average than in Si-rich SROAS. As assessed from the shape of N_2_ sorption isotherms (Fig. 6C), Al-rich (2.6) SROAS appeared as a non-porous solid^59^. Nitrogen sorption experiments accorded with TEM observations and confirmed spatial matter segregation in Si-rich SROAS at scales < 100 nm, offering porosity and considerable SSA. On the other hand, Al-rich SROAS consisted of consolidated particles. Our results demonstrate that nanoscale compaction of synthetic SROAS cannot be reversed by cryomilling as it does not change the magnitude of SSA, limiting the feasibility of a top-down approach to mineral synthesis. Furthermore, structural changes already occurring at short milling durations suggest that prolonged milling is not an option.

### Implications of structure-related aggregation effects for the reactivity of SROAS in the environment

A comparison of spectroscopic properties of synthesized SROAS with natural analogues supports structural similarity. As assessed from Si speciation and elemental analysis, Al-rich SROAS resembled proto-imogolite phases precipitated from waters percolating shales and volcanic rocks (designated as Derbyshire allophane and Ki–P)^51,84^. Furthermore, FTIR spectra indicated a similar composition to Al-rich (Al:Si > 2.8) samples isolated from Podzols^10^. An Si-rich SROAS separated from andesitic ashes exhibited structural similarity to our synthetic Si-rich SROAS with respect to Al:Si ratio, Si speciation and content of Al^IV^ (designated as Okamoto allophane)^29,51^. Specific surface areas of natural SROAS determined by N_2_ sorption ranged from 21 to 478 m^2^ g^−1^^70,85–87^, whereas isotherms indicated the presence of micropores^87^. Values of SSA of naturally precipitated SROAS are thus in the same range as observed for synthetic samples, but as Al:Si ratio and structural properties were scarcely characterized, the relation of composition to SSA in natural samples remains ambiguous.

However, irreversible aggregation is a well-known phenomenon in both bulk soil containing SROAS and particle-size fractions. Andic soil material was irreversibly compacted upon water removal^88^ and lost substantial SSA compared to a treatment without capillary stress^89,90^. Freeze-drying of clay fractions from an andic subsoil caused the formation of micrometre-sized aggregates (> 20 µm)^91^ and aggregation of clay-sized particles was also observed to be caused by air-drying^92^. Saturation deficits at ambient temperature and relative humidity are sufficient to induce a decrease in water retention capacity^93^ and sorption capacity of ionic species to SROAS^80,94^, evidencing physical reorganization to affect the accessible surface. Both evaporation of water and physical processes occurring during freezing lead to a spatial concentration of mineral mass, which causes particles to interact. The dehydration efficacy likely depends on the drying method, whereas it may be higher in freezing compared to air-drying at ambient temperatures.

In soils and sediments, the water regime determines the degree of SROAS dehydration. Formation of SROAS requires sufficient silicate weathering that intensifies under a udic moisture regime. However, SROAS aggregation is enhanced by drying so that aggregation is likely most pronounced in soil with fluctuating water contents. Such conditions prevail for example in sandy substrates, where drying can progress into the subsoil. Precipitation of Al-rich SROAS led to induration of subsoil horizons by the formation of continuous coatings of SROAS with Al:Si ratio of about 2 in andesitic deposits^95^ and podzolic soils^96,97^. Characterization by FTIR spectroscopy evidenced a coating with Si in Q^0^(3Al) conformation^96^. Based on our results, we expect such strongly dehydrated Al-rich precipitates to be relatively unreactive, as aggregation likely decreased accessibility of surface functional groups. Association of SROAS with SOM interferes with physical processes of aggregation and particles assembled less efficiently in topsoils upon drying^92^. SROAS separated from a soil horizon that was characterized by SOM accumulation was structurally different from our precipitates regarding Al speciation, evidencing molecular interactions between SOM and SROAS that affected structure and potentially masked aluminol groups^33^. Besides structure, the water regime and presence of SOM should thus be included in studies on physicochemical mechanisms of SROAS aggregation.

## Conclusion

We synthesized SROAS with variable Al:Si ratios that exhibit structural features similar to poorly crystalline phases precipitating in soils and weathered volcanic ejecta. As we employed ambient conditions, precipitates did not develop a ball- or tube-like morphology and thus likely had a functional group distribution similar to natural nanoscale initial weathering products. We propose that aqueous suspensions contained small polynuclear species that resembled precursors of Al- and Si-rich imogolite and allophane. Our results indicate that composition and crystallinity severely alter the accessibility of functional groups due to changes in aggregation mechanisms during dehydration. Incorporation of Si led to an increase in specific surface area, potentially evidencing a physicochemical impact on SROAS reactivity. Studying physical processes causing aggregation of poorly crystalline minerals as a function of structural properties may thus improve our understanding of their adsorption behaviour in the natural environment. Retention of organic substances by SROAS is particularly relevant for carbon sequestration in soils and sorption of nutrients, i.e. phosphate, which affects their bioavailability. With respect to future syntheses, we recommend careful customization of dehydration conditions.

## Materials and methods

### Precipitation of SROAS

We synthesized SROAS of variable Al:Si ratios at ambient temperature and pressure by neutralizing a 0.2 M aluminium chloride hexahydrate solution (AlCl_3_; AppliChem GmbH, Darmstadt, Germany) with sodium orthosilicate (Na_4_SiO_4_; abcr GmbH, Karlsruhe, Germany). Due to the pH conditions of the stock solutions, both reactants were in monomeric form, i.e. Al in hexacoordinated, partially hydrolysed, aquacomplexes (pH < 3)^53^ and Si as monomeric silicic acid and dissociated silicate ions^98^. After mixing the stock solutions, the batch volume contained 67 mmol Al and 33, 44 or 67 mmol Si, resulting in molar Al:Si ratios of 1, 1.5 and 2, respectively. Synthesis procedures differed between variants with respect to the course of neutralization. Each synthesis procedure was carried out in triplicate. Titration data is presented in Supplementary Fig. S8. A Si-rich SROAS (Al:Si = 1) was precipitated by mixing 1 l of a 0.2 M AlCl_3_ solution with 2 l of a 0.1 M Na_4_SiO_4_ solution under vigorous stirring within approximately 1 min. The AlCl_3_ solution was acidified with 70 ml of a 1 M HCl solution (Chemsolute, Th. Geyer GmbH & Co. KG, Renningen, Germany) beforehand to keep synthesis pH < 7. The final suspension had a pH of 6.6 (± 0.3; glass electrode, InLab Expert Pro, Mettler Toledo, Gießen, Germany) and was stirred for 1 h, during which the pH increased slightly (+ 0.2). An Al-rich SROAS (Al:Si = 2) was synthesized by mixing 1 l of a 0.2 M AlCl_3_ solution with 1 l deionized H_2_O and 1 l of a 0.1 M Na_4_SiO_4_ solution. The resulting suspension had a pH of 3.8 (± 0.04) and was further neutralized with 234 (± 2) ml of 1 M NaOH (1.17 mol OH^−^ mol^−1^ Al^−1^, AppliChem GmbH) within 30 min. Final pH was 6.1 (± 0.1) and stable during a further 30 min of stirring. Another SROAS (Al:Si = 1.5) was precipitated by mixing 1 l of a 0.2 M AlCl_3_ solution with 2 l of a 0.067 M Na_4_SiO_4_ solution, resulting in a suspension with pH 4 (± < 0.1) that was neutralized (pH 6.4 ± 0.2) with 131 (± 2) ml 1 M NaOH (0.66 mol OH^−^ mol^−1^ Al^−1^). The pH was constant during another 30 min of stirring. Suspensions were subsequently transferred to dialysis membranes with a molecular weight cut-off of 14 kDa (Viskase GmbH, Köln, Germany) and dialysed for one week until the electrical conductivity in the permeate was below 5 µS cm^−1^ for 24 h.

### Retrieval and cryomilling of the solids

As separation by centrifugation and filtration was not practicable, we decided to achieve mass retrieval by freeze-drying. After dialysis, about 400 ml of suspension volume were filled into polyethylene bottles and frozen at − 20 °C in a conventional freezer. Shortly after complete freezing, the suspensions were freeze-dried and pooled per Al:Si variant. Three bottles of frozen Al-rich SROAS suspensions (initial Al:Si ratio = 2) were excluded from this procedure and thawed at room temperature. Rapidly settling precipitates were decanted, pooled and dried at 40 °C. A portion of each variant of freeze-dried material was ground with a ball mill under constant cooling with liquid N_2_ (CryoMill, Retsch, Haan, Germany). About 3 g were filled into the sample chamber, which was then precooled for 3 min and subsequently shaken at a frequency of 30 Hz. Samples did not come into contact with liquid N_2_. We chose a shaking time of 2 min for Si-rich SROAS and 5 min for both Al-rich SROAS.

### Characterization of unground SROAS

Morphology of the synthesized SROAS was studied by ESEM under low vacuum and acceleration voltage of 30 kV (FEI Quanta 250 ESEM, FEI Company, Hillsboro, United States). Their elemental composition was determined by microwave digestion of 0.1 g in 28 ml deionised water, 2.5 ml 68% HNO_3_ and 0.5 ml 40% HF. Aluminium, Si and Na concentrations were quantified by inductively coupled plasma optical emission spectrometry (ICP-OES; Agilent 5110, Agilent Technologies, Waldbronn, Germany). Mineral structure was investigated by XRD, FTIR, ^27^Al and ^29^Si NMR spectroscopy. Mineral powders prepared by grinding in a mortar were exposed to Co Kα radiation (λ = 0.179 nm), and diffraction patterns were recorded from 3 to 60° 2θ at a step size of 0.05° 2θ and a counting time of 1.5 s (D500, Siemens, München, Germany). We obtained FTIR spectra at wavenumbers from 4000 to 500 cm^−1^ with the transmission accessory of a LUMOS infrared microscope (Bruker, Ettlingen, Germany). Pellets were prepared from mixtures of 1 mg of ground sample and 200–280 mg of KBr. Fifty background spectra were recorded against the atmosphere, and subsequently 100 sample scans accumulated at a resolution of 4 cm^−1^. Spectra at wavenumbers from 600 to 270 cm^−1^ were measured with a Nicolet 5700 spectrometer accumulating 64 scans at a resolution of 2 cm^−1^ (Thermo Scientific, Darmstadt, Germany). Pellets were prepared at the same mass ratio, but with a hydraulic press. The chemical environment of ^27^Al and ^29^Si nuclei was studied by solid-state NMR spectroscopy with direct-polarization (DP) and magic angle spinning (MAS) on a 400 MHz Bruker Avance II + spectrometer, equipped with a 4 mm H/X MAS probe. Samples were spun in 4 mm zirconia rotors. ^27^Al NMR spectra were obtained at a resonance frequency of 104.3 MHz and a MAS frequency of 12 kHz. A DP pulse of 22° was applied and relaxation delay was set to 4 s. ^1^H was decoupled with a tppm20 pulse sequence. Between 64 and 256 scans were accumulated. Chemical shifts are expressed relative to an aqueous solution of AlCl_3_. ^29^Si NMR spectra were recorded at a resonance frequency of 79.5 MHz and a MAS frequency of 8 kHz. Decoupling of ^1^H was achieved with a spinal64 pulse sequence. A number of 2127–3072 scans were acquired with DP by a 22° pulse (2 µs) and a delay time of 120 s. ^29^Si spectra were referenced to tetramethylsilane. All data was collected at an acquisition time of 0.04096 s, 4096 observation points and a spectral width of 50 kHz, giving a resolution of 12.02 Hz. Phase adjustment and baseline correction was conducted with TopSpin 4.0.7 (Bruker). The quantitative contribution of Si nuclei in Q^0^(3Al) coordination was derived from fitting Lorentzian functions to spectral data and calculating the proportion of integrals to the total area of the resonance signal (OriginPro 2020, OriginLab Corporation, Northhampton, USA). Spectra were normalized and base line was set to the median of all data points. The position of the resonance signal of ^29^Si in Q^0^(3Al) coordination was fixed to the maximum of the visible peak at − 79 ppm, whereas no specifications were made for other nuclei. Resonance of these ill-defined species was reproduced by 3–5 functions with no specification of peak position, height or width (see Supplementary Fig. S9). We first analysed the data at a line broadening > 0 Hz to identify positions of local maxima and then repeated the fit with spectra at a line broadening of 0 Hz to obtain integrals. We modelled ^27^Al NMR spectra at a line broadening of 0 Hz with a combination of Gaussian and Lorentzian functions, whereas neither peak position nor width or height was fixed (see Supplementary Fig. S10). Integrals of functions were ascribed to a coordination of Al according to the chemical shift^49^ and summed.

We determined the SSA by N_2_ sorption to freeze-dried, unground SROAS manometrically at eleven points below a relative pressure of 0.35 with an Autosorb AS 1 at − 196 °C (Quantachrome Instruments, Boynton Beach, USA) after degassing the samples at 80 °C until the pressure increase was < 0.067 mbar min^−1^. Volume was recorded after 5 min of equilibration. Measurements were replicated three times. A sample of Al-rich SROAS obtained by decanting was analysed at 10 points after 2 min equilibration time on a Quantachrome Autosorb iQ (Anton Paar Quanta Tec Inc., USA) after degassing at 40 °C for 24 h^67^. Specific surface area was derived by linear regression with the Brunauer–Emmett–Teller (BET) equation after selecting data points in the range of continuous increase in sorbed N_2_ times (p°–p) with p:p°^59^.

### Characterization of cryomilled SROAS

Structural analysis by FTIR and NMR spectroscopy was conducted as described above. Specific surface area and porosity were analysed by determining both N_2_ sorption and desorption isotherms on a Quantachrome Autosorb iQ after degassing at 60 °C for at least 8 h. Specific surface area was calculated as described above. Total pore volume (V_T_) was obtained from the volume of sorbed N_2_ at p:p° = 0.99. Respective volumes of pores with a diameter in the mesopore range (2–10 nm, 10–50 nm) were derived from the desorption branch according to the Barret–Joyner–Halenda (BJH) method^83^. To visualize particles by TEM, a small portion of mineral material was dispersed in 98% ethanol, deposited on carbon grids and dried protected overnight at room temperature to remove the excess liquid before insertion in the UHV imaging chamber. Images were recorded with a FEI Tecnai G2 (FEI Europe B.V., Biedermannsdorf, Austria) with 160 kV acceleration voltage.

## Supplementary Information

Supplementary Information

## Data Availability

The datasets generated during the current study are available from the corresponding author on reasonable request.
