# Supplementary material for: Synthesis of short-range ordered aluminosilicates at ambient conditions
Source: Sci Rep. 2021 Feb 18;11:4207. doi: 10.1038/s41598-021-83643-w (PMC7892816; doi:10.1038/s41598-021-83643-w)
Supplement: Supplementary file 1 — Supplementary Information [file 41598_2021_83643_MOESM1_ESM.pdf]

Supplementary information

Synthesis of short-range ordered aluminosilicates at ambient conditions

Katharina R. Lenhardt[a*], Hergen Breitzke[b], Gerd Buntkowsky[b], Erik Reimhult[c], Max Willinger[c], Thilo Rennert[a]

[a] Fachgebiet Bodenchemie mit Pedologie, Institut für Bodenkunde und Standortslehre, Universität Hohenheim, Emil-Wolff-Str. 27, 70599 Stuttgart, Germany

[b] Eduard-Zintl-Institut für Anorganische und Physikalische Chemie, Technische Universität Darmstadt, Alarich-Weiss-Str. 8, 64287 Darmstadt, Germany

[c] Institut für Biologisch inspirierte Materialien, Universität für Bodenkultur Wien, Muthgasse 11/II, 1190 Wien, Austria

**Corresponding author:*

Katharina R. Lenhardt, katharina.lenhardt@uni-hohenheim.de

Contact information:

Hergen Breitzke, breitzke@chemie.tu-darmstadt.de

Gerd Buntkowsky, gerd.buntkowsky@chemie.tu-darmstadt.de

Erik Reimhult, erik.reimhult@boku.ac.at

Max Willinger, max.willinger@boku.ac.at

Thilo Rennert, t.rennert@uni-hohenheim.de

Table S1: Specific surface area (SSA) of short-range ordered aluminosilicates quantified by linear regression with the Brunauer-Emmett-Teller (BET) equation (see method section). Table S1 presents parameters used to calculate monolayer capacity (n_{mon}), C and SSA. Sample mass (m) corresponds to sample mass after outgassing.

Al:Si	Sample treatment	m [g]	Intercept	Std error	Slope	Std error	R^2	n_{mon} [mol g^{-1}]	C	SSA [m^2 g^{-1}]
1.4	unground	0.6985	2.35	0.22	327.83	1.22	0.99	0.00303	140.6	295.5
	cryomilled	0.2498	3.45	0.12	352.33	0.81	0.99	0.00281	103	274.2
2.1	unground	1.4499	34.61	0.72	3783.61	4.28	0.99	2.62×10^{-4}	110.3	25.6
	cryomilled	0.2802	91.72	5.68	2168.29	25.71	0.99	4.42×10^{-4}	24.6	43.2
2.6	unground	1.382	18923.33	1207.58	128222.97	5796.30	0.96	6.80×10^{-6}	7.8	0.7
	decanted, unground	1.3848	12060.39	231.07	39465.19	1184.27	0.99	1.94×10^{-5}	4.3	1.9
	cryomilled	0.3057	48.97	2.81	14092.77	52.17	0.99	7.07×10^{-5}	288.8	6.9

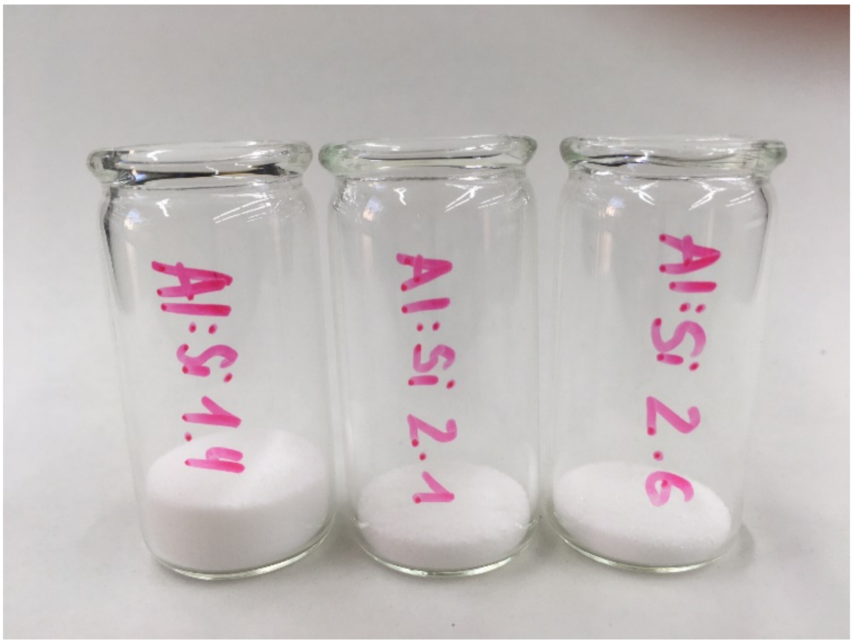

Figure S1: Samples with a mass of 1 g each of freeze-dried short-range ordered aluminosilicates.

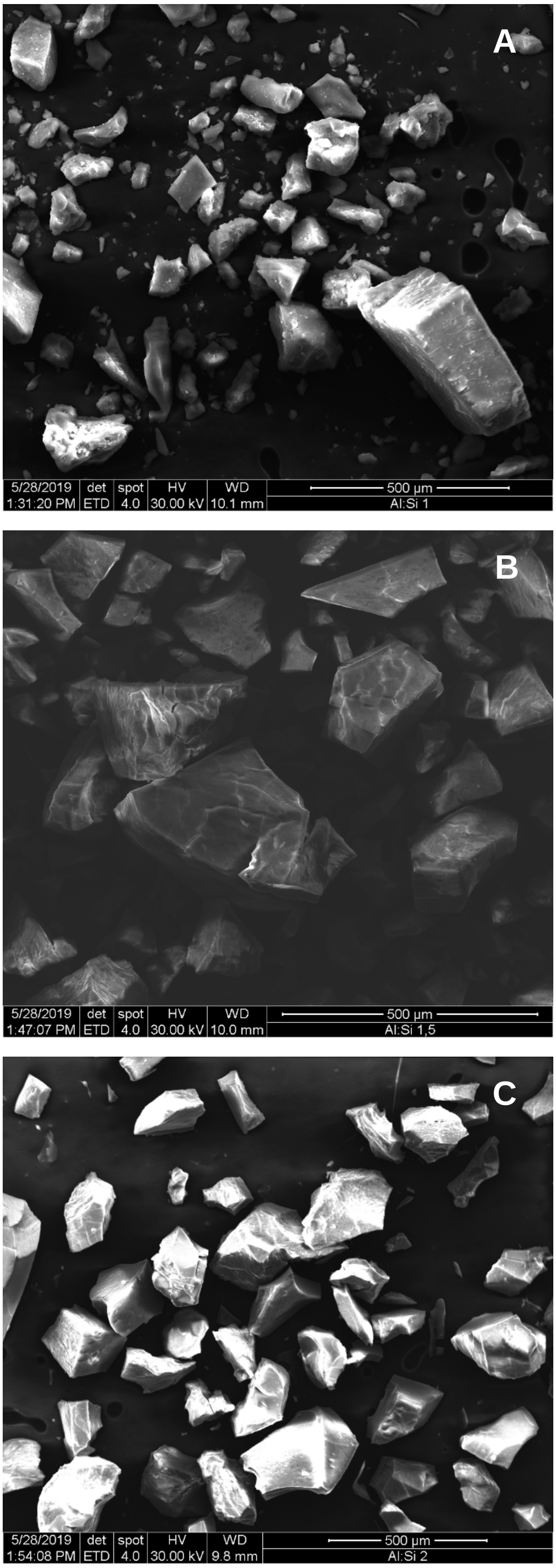

Figure S2: Morphology of aggregates of short-range ordered aluminosilicates with Al:Si ratios of 1.4 (A), 2.1 (B) and 2.6 (C) visualized by environmental scanning electron microscopy. The images were taken by Christian Buchmann.

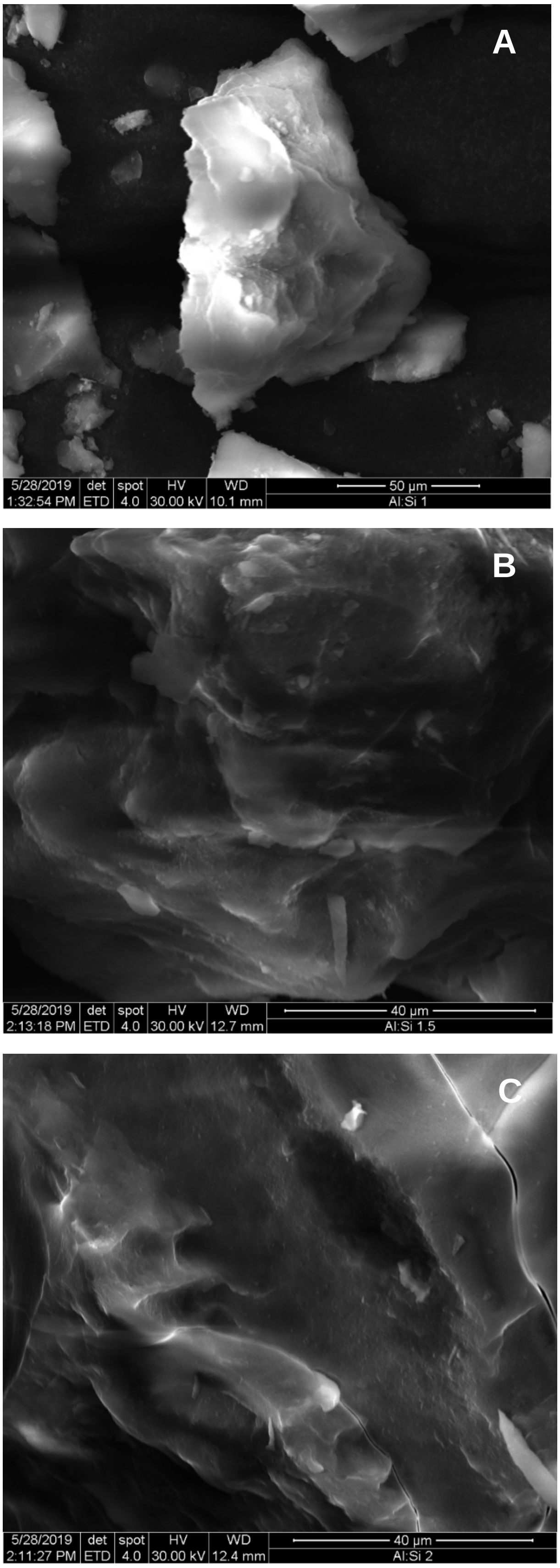

Figure S3: Aggregate surfaces of short-range ordered aluminosilicates with Al:Si ratios of 1.4 (A), 2.1 (B) and 2.6 (C) visualized by environmental scanning electron microscopy. The images were taken by Christian Buchmann.

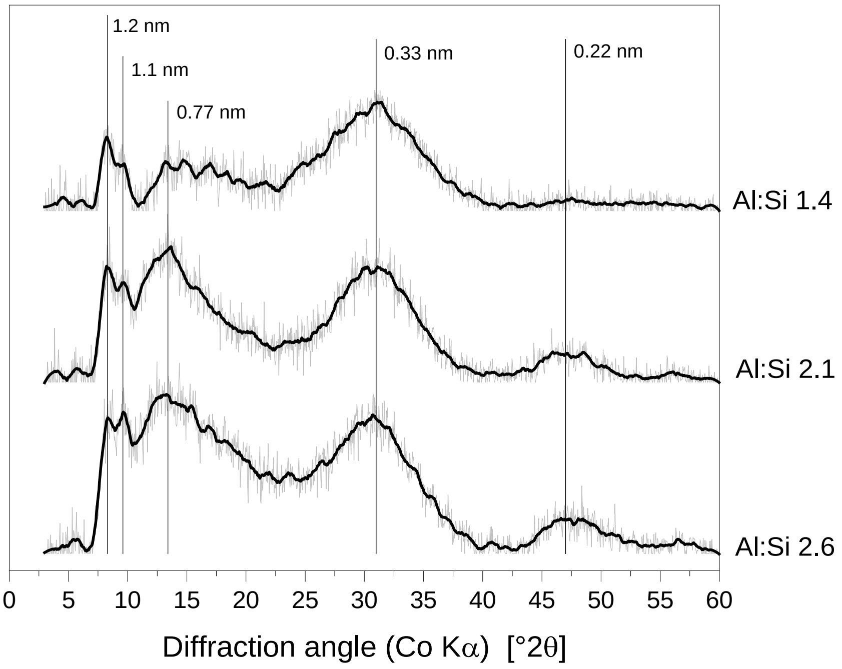

Figure S4: Baseline corrected X-ray diffractograms of short-range ordered aluminosilicates (SROAS). Data is shifted along the y-axis for clarity and given in arbitrary units. Black lines correspond to smoothed datasets. Several local maxima corresponded well with previously published analyses of natural and synthetic SROAS and respective *d*-values are thus delineated.

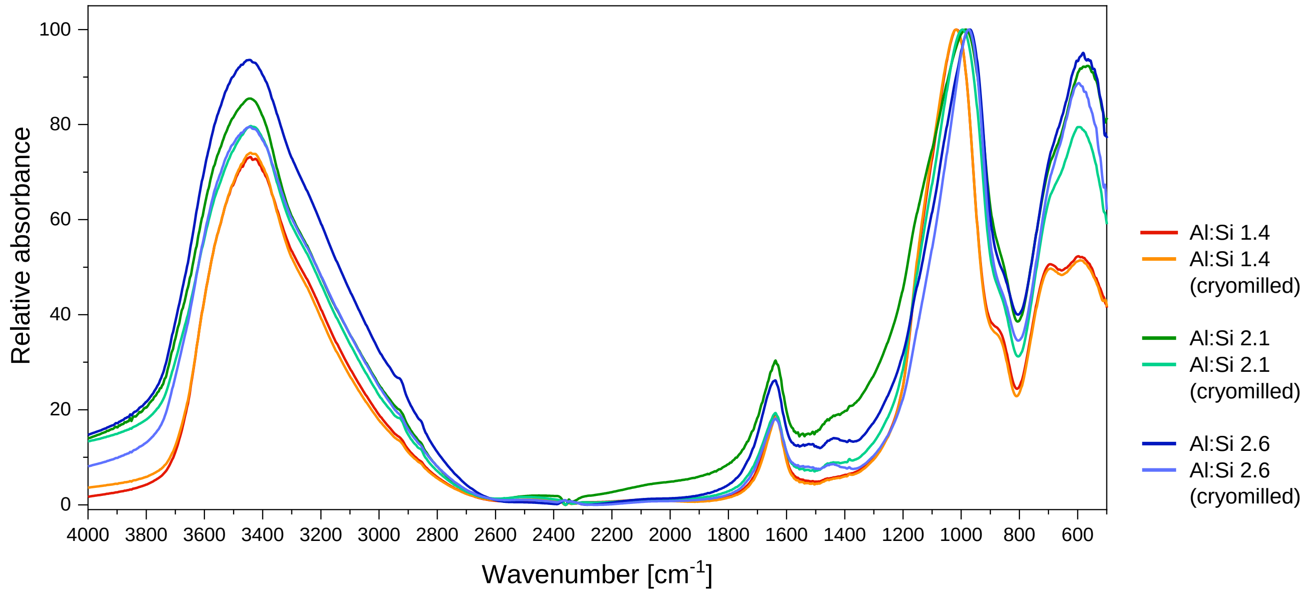

Figure S5: Fourier-transform infrared spectra of short-range ordered aluminosilicates recorded in transmission mode.

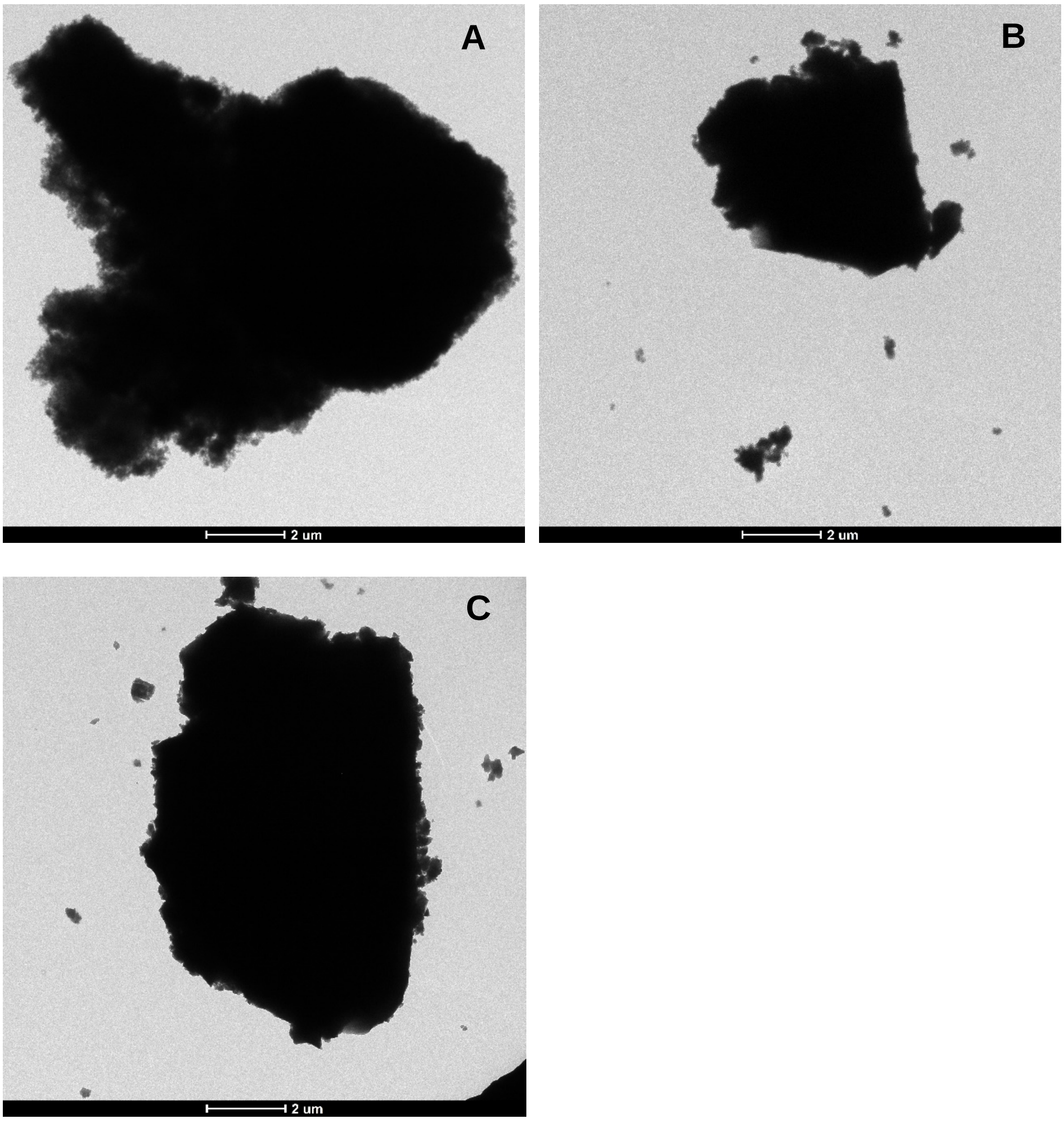

Figure S6: Largest single particles of cryomilled short-range ordered aluminosilicates with Al:Si ratios of 1.4 (A), 2.1 (B) and 2.6 (C) visualized by transmission electron microscopy.

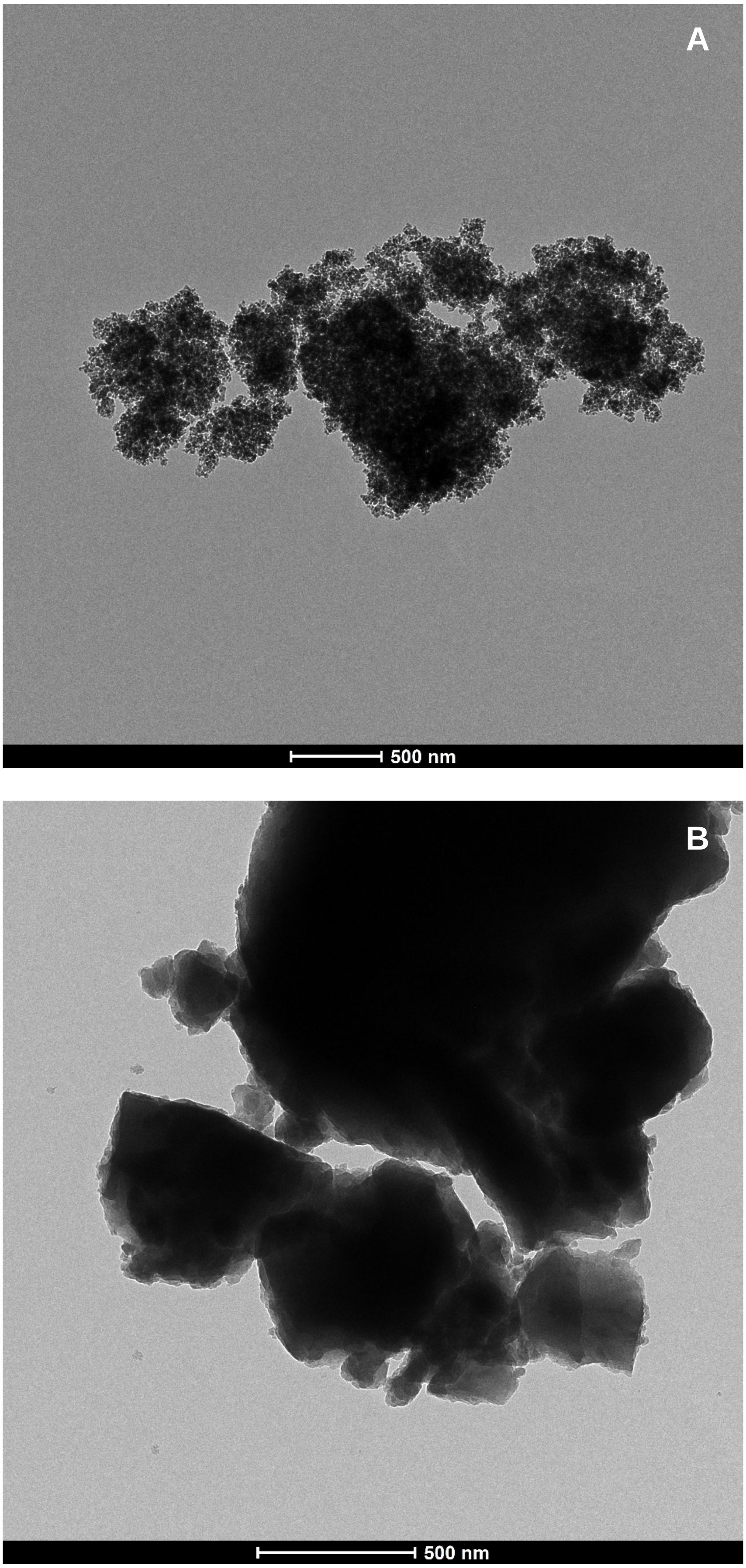

Figure S7: Particle clusters of cryomilled short-range ordered aluminosilicates with Al:Si ratios of 1.4 (A), 2.6 (B) visualized by transmission electron microscopy.

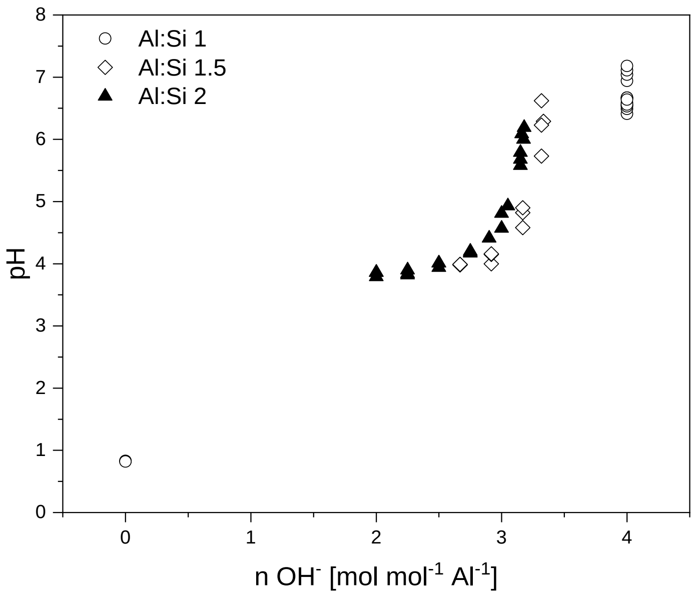

Figure S8: Course of neutralization during synthesis of short-range ordered aluminosilicates (SROAS) as a function of hydroxide added to synthesis batches with different molar ratios of Al and Si concentration. The amount of hydroxide is expressed as a molar ratio of OH:Al, with both aqueous Na_4SiO_4 and NaOH included. The latter was used only for precipitation of Al-rich SROAS (Al:Si 1.5 and 2, see method section). An amount of 4 mol hydroxide was assumed to be released by dissolution of 1 mol Na_4SiO_4. Note that the rate of addition differed between variants and Al supplying solution was acidified by 0.35 mol H mol^{-1} Al^{-1} in the case of Si-rich SROAS.

Peak Analysis

Data Set:[AlSi2lb0basel]AlSi2_lb0_baselinecorr!C   Date:26.01.2021

BaseLine:Constant

Chi^2=1.08168E+01   Adj. R-Square=7.82955E-01   # of Data Points=32767

SS=3.54346E+05   Degrees of Freedom=32759

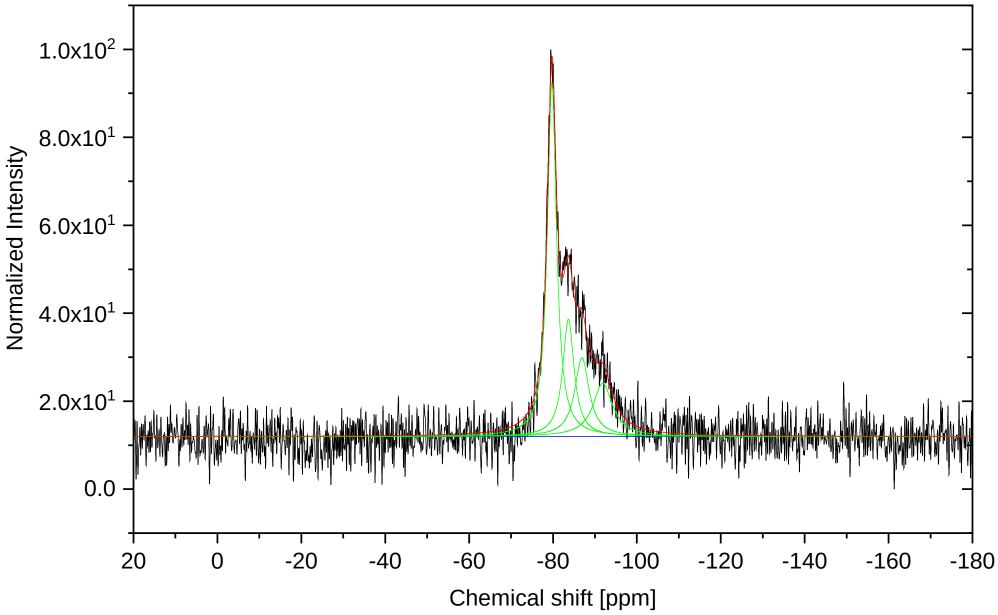

Fitting Results

Peak Index	Peak Type	Area Intg	Area IntgP	Center Grvty	Max Height	FWHM
1	Lorentz	-106.61877	-15.5254	-91.87301	12.11999	5.63265
2	Lorentz	-116.52321	-16.96764	-86.89956	17.93139	4.15449
3	Lorentz	-136.23558	-19.83808	-83.67155	26.67225	3.26252
4	Lorentz	-327.36032	-47.66889	-79.74923	80.51822	2.59512

Figure S9: Peak analysis report of solid-state ^{29}Si nuclear magnetic resonance spectra of short-range aluminosilicates with Al:Si ratio of 2.6. Proportion of Si in Q^0(3Al) (peak centred at -79.7 ppm) was derived from integral contribution ("Area IntgP" [%]; see method section).

Peak Analysis

Data Set:[AlSi2]Al_AlSI2_lb0_baselinecorr!C　Date:07.09.2020

BaseLine:Constant

Chi^2=3.99924E-01　Adj. R-Square=9.97711E-01　# of Data Points=32767

SS=1.30983E+04　Degrees of Freedom=32752

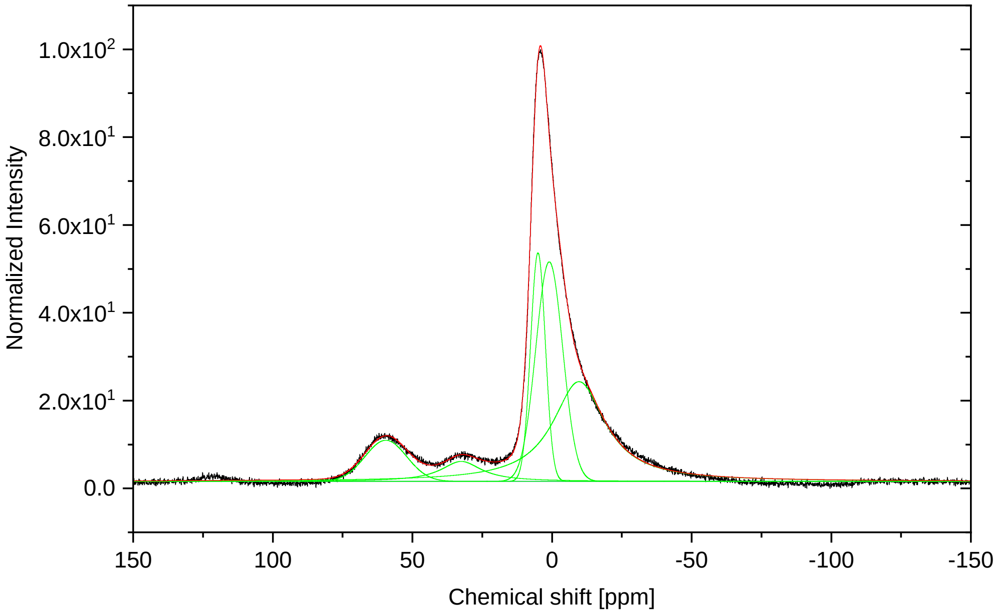

Fitting Results

Peak Index	Peak Type	Area Intg	Area IntgP	Center Grvty	Max Height	FWHM
1	Lorentz	-787.75223	-38.69028	-9.66499	22.71992	22.77551
2	Gauss	-605.20997	-29.72476	0.99142	50.00236	11.37063
3	Gauss	-344.99331	-16.94427	5.02881	52.06778	6.22459
4	Lorentz	-123.20389	-6.05113	32.50543	4.55315	17.63969
5	Gauss	-174.88744	-8.58956	59.50739	9.36922	17.53574

Figure S10: Peak analysis report of solid-state ^{27}Al nuclear magnetic resonance spectra of short-range aluminosilicates with Al:Si ratio of 2.6. Proportion of Al in tetrahedral, pentahedral and octahedral coordination was derived from respective integrals. Content of octahedral Al was calculated from the sum of integrals centred at < 6 ppm (“Area IntgP” [%]; see method section).

Calculation of a hypothetical diameter of primary particles of Al-rich (2.6) SROAS

Calculation of a hypothetical particle diameter based on cubic close packing was done to rationalize the observation of poorly crystalline SROAS assembling to aggregates that are impermeable towards nitrogen diffusion. Hofmann et al. (2004) characterized aggregates formed by freezing suspensions of Fe (hydr)oxide particles at conditions similar to our procedure. Measured porosity and pore sizes corresponded well with values calculated on the basis of cubic close packing of primary particles (Ø 2 nm, determined by TEM). The face diagonal of this arrangement`s unit cell is $4r$ with sphere radius r. Application of Pythagorean law results in an edge length of $2\sqrt{2}r$. The diameter of the void between spheres is $2\sqrt{2}r - 2r$. Based on the assumption that nitrogen diffusion is restricted by pore sizes in Al-rich (2.6) SROAS, we set the void diameter to 2 nm and calculated the respective sphere radius and diameter ($r =$ 2.4 nm, Ø = 4.8 nm). We are aware that the assumptions for this calculation are an ideal approximation of the conditions in Al-rich SROAS, which are in reality modified by, for instance, deviation from monodispersity and particle shape. Nonetheless, the calculations indicate that polynuclear aluminosilicate species with local imogolite-like structure (Si in Q^0(3Al)) have to be small (< 4 nm) to hinder diffusion of nitrogen to interparticle spaces and that surface accessibility is very sensitive to interparticle distances (i.e. effective r), which is determined by the width of the hydration layer and thus the binding mechanism.
